# Evaluation of the primary health care expansion program with public-private partnership in slum areas from the perspective of stakeholders: a qualitative study

**DOI:** 10.1186/s12875-024-02303-w

**Published:** 2024-02-22

**Authors:** Aboalfazl Marvi, Fatemeh Kokabisaghi, Seyed Saeed Tabatabaee, Ehsan Moallem, Javad Moghri

**Affiliations:** 1 Student Research Committee, Mashhad University of Medical Sciences, Mashhad, Iran; 2 Social Determinants of Health Research Center, Mashhad University of Medical Sciences, Mashhad, Iran; 3 Department of Management Sciences and Health Economics, School of Health, Mashhad University of Medical Sciences, Mashhad, Iran

**Keywords:** PHC, Suburban area, Slum areas, Public-private partnership model, SWOT model, Evaluation, Iran

## Abstract

**Background:**

Today, economic and social determinants of health in slum settlements are at the policymakers’ center of attention. Iran has had an excellent experience in the Primary Health Care Program. This study aimed to evaluate the Primary Health Care Expansion Program with public-private partnerships in slum areas of Iran from the perspective of stakeholders in 2022.

**Methods:**

This qualitative study was conducted using the framework content analysis method. Participants were 17 experts and health managers involved in The Primary Health Care Expansion with Public-Private Partnerships in the suburban areas at the medical universities of Khorasan Razavi province in the east of Iran, who were selected through purposive sampling via snowball method. For data collection, a semi-structured interview was done and framework content analysis was used for data analysis and results categories based on the SWOT.

**Results:**

The study identified 23 main themes and 112 sub-themes, which were then grouped into four main categories - strengths, weaknesses, opportunities, and threats using the SWOT model.

**Conclusion:**

Results of the study showed the internal and external factors affecting Primary Health Care Expansion with Public-Private Partnerships in suburban areas. This situational analysis can help health policymakers to better understand the performance of health facilities.

**Supplementary Information:**

The online version contains supplementary material available at 10.1186/s12875-024-02303-w.

## Introduction

Nowadays, slum habitations are among important challenges of developing countries [1]. Improving slum areas have been considered as one of Sustainable Development Goals [2]. United Nations clearly indicated these habitants should not be left behind, particularly with regard to access to healthcare. Moreover, in the Universal Health Coverage (UHC) Strategy emphasizes the importance of ensuring everyone’s access to safe and affordable housing, and life necessities including primary health care (PHC) [3].

Urbanization has been accompanied by increasing the population of slum settlements. These areas are close to large cities and have multiple problems such as overcrowding, poor housing and living environment and low access to safe drinking water and health care services [3]. According to the United Nations World Population Monitoring Report in 2014, 54% of the world’s population lived in cities, which is expected to increase to 66% by 2050. More importantly, about 90% of the change will happen in Asian and African countries [4]. In 2021, based on the report of the World Bank, 73% of Iran’s population lived in urban areas, and it will approximately increase to more than 80% by 2050 [5]. According to the report of the Ministry of Health and Medical Education (MOHME) of Iran in 2015, the population living in the slum areas was estimated to be more than 10 million [6]. Increasing urbanization and immigration to cities especially to slum areas can affect Iran’s health system [7].

Unbalanced and unequal distribution of facilities and infrastructure in slum areas has made access to health services difficult [8]. More importantly, even with physical access to health services, due to poverty, the rate of services utilization is low in these areas [9]. The results of a study in Iran showed that increasing migration from villages to cities and slums was associated with changing the burden of diseases, and unequal access to health services. Moreover, unemployment, and low income might decrease financial access to health services and the health level of people living in these areas [7] Not having a plan to improve the health of slum inhabitants can become a serious threat to the health of whole population [10]. Therefore, the World Health Organization has drawn the attention of governments to these areas [11].

Achieving UHC and dealing with challenges such as environmental, demographic and changing the burden of diseases require specific health service delivery model appropriate to countries’ conditions [12]. Iran’s health network started to provide PHC services in 1970s. Even though, it was successful at the beginning, due to the changes in the society, it did not meet the needs of all people later [13]. Family Doctor and Rural Insurance Programs were launched in 2005 with the aim of increasing access to services, establishing a referral system, creating justice and accountability in rural areas [14, 15]. Moreover, Urban Family Physician Program (UFPP) was implemented as a pilot in Fars and Mazandaran (two large provinces of Iran) in 2013 [16]. Although these programs achieved significant success, they have not been successful enough in the suburban areas [2, 17]. A study in the slum areas of Alborz province in Iran showed that in these areas, access to screening, educational, counseling and health care services was far from optimal, so that respectively, only 26.1 and 24.1% of people in need of nutritional and psychological counseling received their necessary services [1].

To overcome the difficulties and to provide and promote PHC, Health Sector Reform Plan (HSRP) was launched in 2014. The first phase focused on suburban areas and had 6 goals including 1) improving health indicators, 2) health equity, 3) quantity and quality of health services, 4) satisfaction of users and providers, 5) changing the behavior of target population and providers of health services, 6) reforming the payment mechanism and the method of purchasing services with a public-private partnership (PPP) model. In order to supply human resources and provide basic health services in suburban areas, a structure relatively similar to that of rural areas of Iran was applied (a health center for 12,500) [18].

PPP model has been implemented through service outsourcing and performance-based payment. Differences in management, and financial, and human resources, as well as equipment and infrastructures in different provinces, and cultural, social and economic factors have made taking specific interventions and evaluation necessary (2). One of the appropriate models that can be used to evaluate the performance is Strengths, Weaknesses, Opportunities and Threats (SWOT). It helps to analyze the situation of organizations and to identify effective interventions [19]. The present study was conducted with the aim of evaluating the Primary Health Care Expansion Program (PHCEP) in suburban areas of Iran, with the participation of the public-private sector from the perspective of stakeholders.

## Methods

### Study design

This study was conducted with a qualitative approach using the framework content analysis method.

### Participant and sampling

The target participants in the study included 17 experts and health managers from the health centers and vice-chancellors for health at the medical universities of Khorasan Razavi province in the east of Iran. These individuals involved in the Primary Health Care Expansion Program with Public-Private Partnership in slum areas. In this study, we used a purposive sampling via snowball method. The inclusion criteria were having at least 3 years of experience in establishing, managing, or delivering primary healthcare services, as well as an interest in participating in the study. Those who were not willing to be a part of the study were not included.

### Data collection

The semi-structured interviews were conducted to collect data from October to December 2022. The interviews were done by a trained expert and aware of interview techniques. The preliminary interview guide was prepared through a review of the literatures and discussion with research team, following three interviews and discuss with research team, it was further edited and developed. a total of 17 interviews were done face-to-face or via telephone with experts and health managers. The interviews conducted in Persian language at the interviewee’s workplace (15 interviews) or via telephone (2 interviews). The average duration of interviews was 30–60 minutes. Before conducting the interviews, the consent of all participants was obtained. The interviewer presented the study aims for each interviewee and then asked the questions (See Appendix 1). All interviews were recorded via a voice recorder with the consent of the participants. Additionally, during each interview key points, non-verbal signs and facial expressions were noted. Data collection was stopped when the researchers detected that more interviews did not provide them with new data. In other word, data saturation occurred.

### Data analysis

Tow researchers independently carried out the data analysis utilizing framework methods, consisting of five steps included ‘familiarization’, ‘developing an analytical framework,’ ‘indexing’, ‘charting’ and interpreting the data [20]. at the end of each interview, a researcher typed its content word-by-word, key points, non-verbal signs, and facial expressions were taken into account. In the second stage, two researchers independently read the interview texts at least three times for familiarization. In the third stage tow researchers read the interview text line by line carefully to extract meaning unit and create initial codes by the inductive method. In the fourth stage, the researchers grouped the codes into categories based on similarities and differences, and researchers’ viewpoints were obtained and used. In the five stage the main categories were placed in the SWOT framework, and the initial manuscript was prepared in the original language. Ultimately, the original manuscript was translated from Persian to English in the following manner in order to publish the research: The initial translation was done by a research team member (FKS) who has expertise in the study’s field, a deep understanding of both the original and target language, and a familiarity with the participants’ culture and study context. In the next step, the translation was compared with the initial manuscript, especially original themes and participant quotes by researcher – translator (FKS).

Since counseling could be a feasible method for ensuring reliability in the translation of qualitative studies [21], On the other hand, the forward and backward translation may bring about the removal of a portion of the participants’ quotes once the initial text has been translated [22, 23]. ‘To ensure the accuracy of participant quotes, themes, and the selection of appropriate words and phrases, two roundtable discussions were conducted before finalizing the translation and ultimately approved.

### Reliability

Lincoln and Guba’s criteria were used to increase the accuracy and consistency of the results. To increase credibility, participants were selected with maximum diversity. To create reliability, the findings were sent to the participants for confirmation by them. For confirmability research team was involved in all research processes. To create transferability, the research process was presented completely, and the participant’s opinions were cited without bias.

## Results

In this study, interviews were conducted with 17 participants, including University professors, healthcare managers, family physicians, and community health workers, from suburban health services centers and vice-chancellors for health at medical universities of Khorasan Razavi in the east of Iran. Among the participants, 13 were men and 4 were women, with an average age of 43 years. A summary of their demographic characteristics is provided below (see Table 1).

**Table 1:** Demographic information of the study participants

Variables	Items	Frequency
Gender	Male	13
	Female	4
Average age	Male	45
	Female	49
Academic degree	PhD	3
	MD	10
	Master	1
	Bachelor	3
Job experiences (YEARS)	5–10	2
	10–20	12
	20>	3
Positions	University professor	2
	Health managers	4
	Headquarters experts	6
	Family physicians	2
	Community health workers	3

**Fig. 1:**
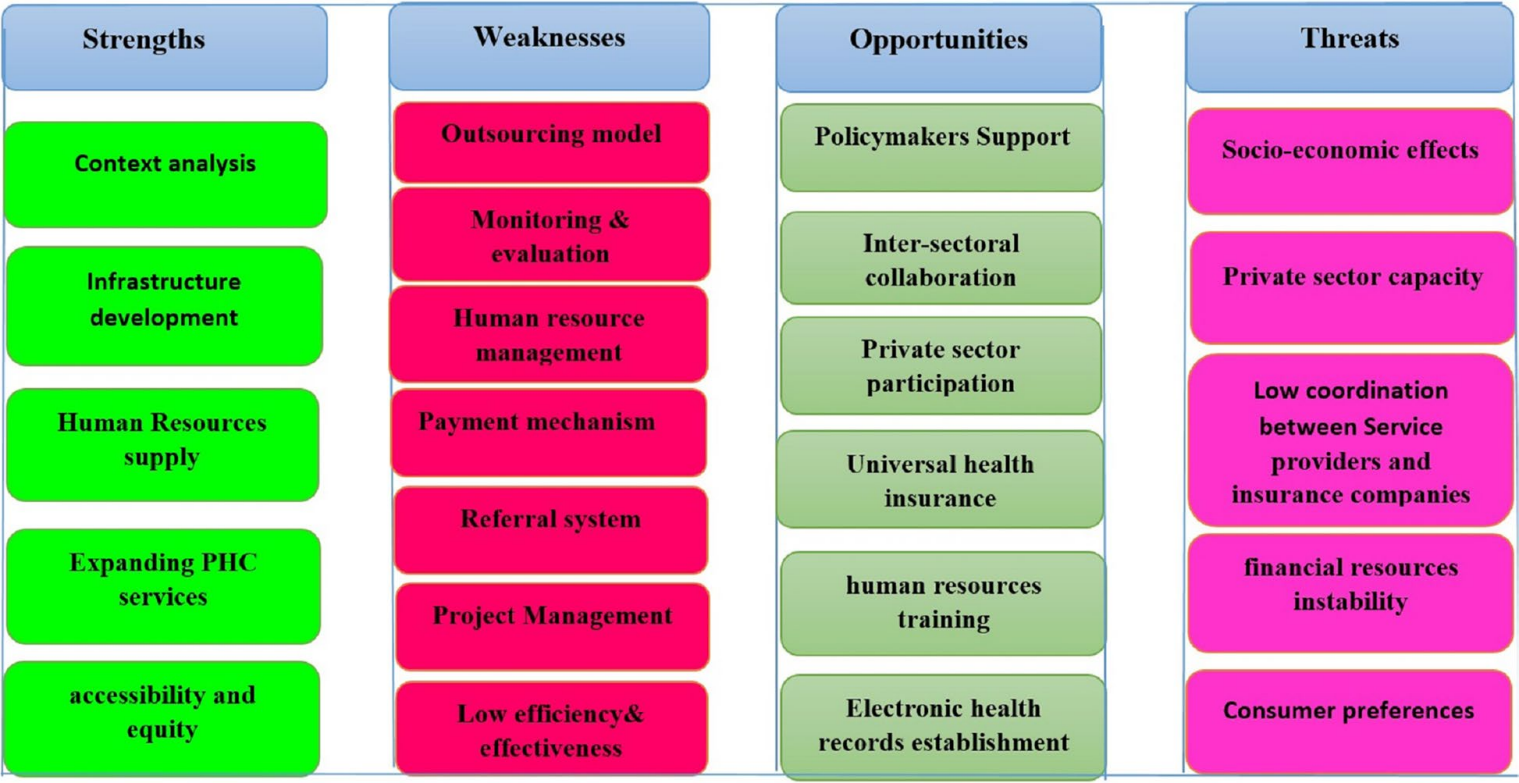
The swot categories and subcategories based on the PHC experts’ viewpoints

In this research, data was presented in 4 main categories including strengths (5 themes, 24 subthemes), weaknesses (7 themes, 43 subthemes), opportunities (6 themes, 27 subthemes), and threats (5 themes, 18 subthemes) (Tables 2, 3, 4 and 5). A summary of SWOT categories and main themes based on the PHC experts’ viewpoints is presented (See Fig. 1).

**Table 2:** Strengths of the Primary Health Care Expansion Program

Themes	Subthemes
Context analysis	Collecting population data
	Getting to know the characteristics of the population in terms of distribution and age groups
	grouping the population and determining the areas covered by the centers
	Preliminary assessment of the population in terms of disease prevalence
	House-to-house census and identification of environmental problems
Development of infrastructure and physical resources	Allocation of resources and development of physical environment
	Building centers and equipping them in slums
	The purchase of electronic equipment to provide health services
	Development of the physical space of health centers
Supply of Human Resources	Recruitment in the form of health teams in slum areas
	Recruitment with the help of the private sector
	Recruitment of nutrition experts and psychologists to the health teams
	Increase in the staff to population ratio
	Recruitment of multi-professional staff in health centers
Strengthening and expanding PHC services	Providing New Health Service (New PHC)
	Providing psychology and nutrition counseling services
	Screening and care of non-communicable diseases
	Providing active care to the population
	Strengthening the infectious disease care system, especially in Covid-19 pandemic
	Promoting health literacy and self-care culture
Increasing access to and equity in providing services	Increasing service coverage in underprivileged areas
	Attention to the principles of primary health care
	Increasing access to services by building new centers

**Table 3:** The weaknesses of the Primary Health Care Expansion Program

Themes	Sub-themes
Outsourcing model in PHC	The private sector does not take the responsibility
	Service outsourcing model is not clear for stakeholders
	Outsourcing implementation method has weaknesses
	Employing with different contract and payment methods
	The outsourcing model is not appropriate
	Not using previous experiences and models implemented in the system
Performance monitoring and evaluation	There is no standard evaluation tool to measure professional performance
	Private companies aim at profit and do not pay attention to performance
	Basis of payment is the service registration. The services have not been properly defined
	Monitoring and evaluation do not affect the salaries which are not based on performance
	Companies do not have the ability to perform specialized monitoring
	Due to the large movement of employees, proper monitoring can’t be done
	Recruitment models and delays in payments impact the evaluation negatively
Human resource management	Incorrect mechanism of recruitment and distribution of human resources
	There is no balance between workload, manpower and pay scale
	Lack of promotion system and job security and the stress of dismissal
	Delays in payment reduce the motivation to improve performance
	Inadequate response to human resources needs
	Lack of amenities and space for providing services
Payment mechanisms	The payment mechanism is not proportional to the type and load of services
	The payment mechanism is not based on performance
	The payment mechanism does not motivate the provision of prevention services
	Using two different payment models in health centers
	Delays in payment of salaries and irresponsibility of the private company
	The payment mechanism is not fair and does not include incentives
Referral system	Weakness in providing patient care due to the lack of communication between level one and two of health network
	Disconnection between electronic patient record systems in private and public sectors
	Weak connection of medical specialists with PHC
	Problems in patient care due to the weak patient referral system
	lack of a proper referral system
Project Management	Implementation of the program without pilot and evaluation phase
	The purpose of outsourcing has gone from productivity to human resources supply
	Legislative challenges
	Unequal distribution of resources
	Focus on the design and implementation methods and not actual performance
	Failure to delegate authority to the private sector
	Not introducing PHC to the target population
Efficiency and effectiveness	Failure to achieve the goals
	Low productivity
	Low efficiency of private employees
	The cost-benefit ratio is low and the resources do not have the necessary efficiency
	Choosing an inappropriate outsourcing model in PHC
	Not using past experiences and opportunities such as the rural family doctor model

**Table 4:** Opportunities of the Primary Health Care Expansion Program

Themes	Sub-themes
The support of policymakers and the law	Legal support for the program
	Health sector reform documents and guidelines
	Detailed guidelines on the implementation requirements and allocating resources
	Support of Sustainable Development Program
Inter-sectoral collaboration	The capacity of health organizations and partners
	Development of health infrastructure with inter-sectoral cooperation
	Construction and development of centers with the cooperation of stakeholders
	Supporting vulnerable groups
	Collaboration of educational and religious institutions in health education
Private sector participation	The capacity of the private sector
	Collaborating with the private sector
	Removing shortages in resources with the support of the private sector
	Delivering new packages of services with the help of the private sector
	Using the capacity of the private sector in crisis such as the Covid-19 pandemic
Universal health insurance	Insurance coverage of the target population
	Reducing people’s health expenses
	Facilitating screening and care in level one healthcare
	Cooperation of patients in referral due to being insured
Adequate and trained human resources	Universities’ educational capacities in the province
	Availability of human resources
	Plenty of job applicants
	Support of the network in training new staff
Electronic patient record and service registration systems	Electronic information system for managerial decisions
	Access to up-to-date indicators and information
	Facilitating the process of patient care and follow-up
	Reducing errors and the workload of the health teams

**Table 5:** Threats to the Primary Health Care Expansion Program

Themes	Sub-themes
Increasing slum habitation and its socio-economic effects on health	The increasing number of slum population
	The increase in migrations to cities and the problem of tracking systems
	Problems of legal and illegal immigrants about necessary services and health insurance
	Different lifestyle in slum areas
	Social problems related to health
Low capacity of the private sector	Weak financial ability of private companies seeking collaboration with health network
	Companies’ lack of awareness of health programs and regulations
	Lack of expertise in monitoring the performance of human resources
Discrepancy between service providers and insurance companies	Differences of insurance companies in providing health services
	Parallel programs
	Disruption in providing services to patients
	Appointing a doctor by the insurance company for insurers
Instability of financial resources	Delay in allocating or distributing financial resources
	Instability of health financial resources
	Complaints about delays in payments
The culture of preferring medical specialists to PHC providers and low awareness on and use of PHC services	People’s willingness to visit specialists instead of PHC
	Low acceptability of PHC services among people
	People’s low awareness of the services provided in PHC and their benefits
	Changing health needs of society

### Strengths

The participants pointed out several strengths of PHCEP shown in Table 2 and explained below.

#### Context analysis

One of the strengths of PHCEP in suburban areas was the better understanding of the environmental and demographic situation of the target population. In this regard,

*“One of the good things that was done at the beginning of the program was establishing a data system about the population living in slum areas. Now, we know the structure of the population and the centers.”(University professor, P1, Inteview1)., “At the beginning, to know the population, we collected data about the population and the areas covered by each health center.”(*CHW*, P14, Interview14).*

#### Development of infrastructure and physical resources

One of the strong aspects of the program was the development of physical space and equipment for providing health services that resulted in improving people’s access to services and the quantity and quality of services. According to,

*(P9), “One of the strengths of the Health Reform Plan and the strengthening of primary health care was that we were able to develop our facility centers. We did not have a center in many suburban area “. (University professor, P9, Inteview9), “I firmly say that before the implementation of this program, we did not have the equipment to provide services in many of our marginal centers (Health manager, P2, Interview2).*

#### Supply of human resources

One of the positive points of the expansion program was the provision of human resources that was able to solve some of the challenges of providing preventive services in suburban areas.

*“In this program, new health care workers, midwives and doctors were recruited, as well as nutrition experts and psychologists that we didn’t have them at all”(University professor, P1, Interview1), “One of the valuable aspects was that our employees were doubled, and very motivated young people came to work here”(Health manager, P12, Interview12).*

#### Strengthening and expanding PHC services

With the implementation of the program in suburban areas, the services provided at the first level were developed and expanded. New PHC service including nutrition counselling and mental healthcare were added to the service packages, and the previously established services such as non-communicable disease screening were strengthened.

*“New programs including nutrition and psychological services were added to the system, and many services were developed with new resources.”(Health Expert, P,10, Interview10) “The implementation of this program made 50% of our centers that were not active, become active”(University Professor, P9, Interview). “One of the important things is that the services became activated, and the ignored services received attention”(CHW, P14, Interview).*

#### Increasing access and equity in providing services

One of the strengths of the program was enhancing equity in the access of slum population to health services.

*“We didn’t have strong PHC centers in the peripheral areas; we got resources, then we were able to build centers, and we improved access”(University Professor, P1, Interview P1). “Access has improved in the city especially in the suburban”(CHW, P14, Interview). “The centers in the slums made significant access, I know and see this clearly, and the quality of services increased. We provide many services that people need and cannot afford, such as mental healthcare.”(Health Expert, P4, Interview 4).*

### Weaknesses

The participants pointed a number of weaknesses for PHCEP showed in Table 3 and explained in below.

#### Outsourcing model in PHC

One of the important aspects of the program was the participation of the private sector and the use of the capacities of this sector. However, according to the participants in this study, outsourcing has not been done with an appropriate model.

*“In principle, it was a good plan, but we requested to implement it like rural FDP. We had the experience, but we signed a contract with a private company. I think we could have saved a lot of resources by using our staff.” (Health manager, P12, Interview12), “We didn’t need a company that takes the money from us as an intermediary to pay the staff. We should have asked a private doctor in the area to provide the package of services”(University professor, P9, Interview9). “The experience of privatization was very good; if it was implemented correctly, it could increase productivity, but we did not do this, and our goal was to eliminate the lack of resources; if we did it correctly, it would be good.” (Health Expert, P10, Intreview10).*

#### Performance monitoring and evaluation

One of the challenges of the PHC system is the evaluation of programs.

*“Now, I have signed a contract with a company and I asked them to monitor the system from a technical point of view. They do not know what to do at all. Moreover, the existing checklists monitors the executive and management processes, and not the technical aspects”(Health manager, P11, Interview 11). “The company is an intermediary in the provision of services, and it is not involved in monitoring and has no manpower for evaluation.”(Health Expert, P8, Interview8) “We were supposed to pay according to the performance. Now that the labor law must be respected, I no longer have the means to manage”(University professor, P9, Interview 9).*

#### Human resource management

One of the important resources of any organization is human resources, which plays an important role in improving the performance and productivity of any organization. In the expansion of health network, the provision of human resources was considered, but the recruitment, distribution and performance monitoring models still are challenges.

*“One of the main problems was private and public employment. With different conditions, employees lost motivation.”(University professor, p1, Interview1) “One of the problems was the unfair distribution of manpower between peripheral and non-peripheral centers. (Health expert, P4, Interview4)”.*

#### Payment mechanism

One of the important management tools in the successful implementation of programs, improving quality of services and manpower’s motivation is the payment mechanism. The payment model for the health team is problematic according to the study participants.

*“In my opinion, the current payment model reduced the motivation of the employees who are Achilles’ heel of the system”(University professor, p1, Interview1). “One of the problems of this program was that the performance measurement units were the services. But the payments were not according to them”(CHW, P14, Interview 14). “The personnel are paid as before and they are not motivated because it has not made a difference”(family physician, P6, Interview6). We paid according to labor law; it would be good if we considered incentives too”(Health expert, P10, Interview10).*

#### Referral system

Referral system is one of the missing chain links of health system; if it is implemented correctly, it can solve many problems.

*“We run screening programs very well, now we should send people to secondary healthcare facilities for diagnosis and we don’t have good communication with other healthcare providers”(Family Physician, P6, Interview6). “One of the problems of the referral system is that our electronic systems must be connected with the private sector”(University professor, P1, Interview1).*

#### Project management

Management is the most important factor in the success of a program, and if the main tasks of management, including planning, organization, coordination, leadership and control, are done correctly, the program will reach the goals. The participants were not satisfied with the management of the program.

*“The program started without a consensus among headquarters, which could help implementing it better (Health Expert, P2, Interview2). “We said it from the beginning to let each province implement the program according to its conditions. We had rural FDP in place and could save resources with continuing that model”(Health manager, P12, Interview12*

#### Low efficiency and effectiveness

One of the important indicators for evaluating the performance of health programs is measuring efficiency and effectiveness.

*“The plan does not have adequate efficiency; the payment model and the type of contracts are exactly the same as the public sector and the indicators are not much different”(Family physician, P6, Interview6). “We privatized to employ the capacity of the private sector to increase productivity, however, because we didn’t have set related goals, I don’t think we did a good job”(Health Expert, P10, Interview10). “Although this project had good benefits, it placed a heavy financial burden on the government.”(Health Expert, P17Interview 17).*

### Opportunities

The participants pointed a number of opportunities for PHCEP showed in table 4 and explained in below.

#### The support of policymakers and the law

The key to the success of the HSRP is the support of policymakers and legal approvals. The implementation of the HSRP resulted in a heightened focus on developing marginalized areas for policy makers, presenting a favorable opportunity to allocate resources and coordinate efforts across sectors. The participants mentioned PPP as an opportunity.

*“The support of policymakers in implementing HSRP in underdeveloped areas is valuable”(University professor, P1, Interview 1). “The legal support of the plan caused the responsible organizations at the provincial level to collaborate with us”(University professor, P9, Interview9).*

#### Inter-sectoral collaboration

*One of the principles of PHC is inter-sectoral collaboration, which is necessary in providing health services.*

*“One of the strengths of this plan was the collaboration of health partners”(Health Expert, P15,Interview15). “The participation of health donors was admirable, wherever we opened a center, donors hepled us.”(CHW,P5,Interview5).*

#### Private sector participation

Using the capability of the private sector in the implementation of health programs is helpful in removing shortages of resources. The interviewees called the PPP model in PHC services as a new experience.

*“We used the resources of private sector to employ personnel, since we couldn’t employ them directly.”(Health manager, P13, Interview 13). “With all the challenges that the program had, if private resources were not used and these personnel did not come, we would be in trouble during the covid-19 period.”(Health manager, P12,Interview12).*

#### Universal health insurance

One of the goals of health systems is financial protection of population against catastrophic health expenditures. In HSRP, the universal insurance coverage program was implemented. The participants called it as an opportunity.

*“One of the important aspects of this program was universal health insurance, which helped a lot in providing care”(University Professor, P1, Interview1). “Universal insurance helped the covered population to receive services.”(Health Expert.P15, Interview15).*

#### Adequate and trained human resources

One of the important resources of any organization is competent and trained human resources. The participants said that one of the positive aspects of this plan was the availability of manpower.

*“Employing a group of trained motivated young people was an opportunity”(Health manager, P12, Interview12). “The collaboration of all universities in training human resources is valuable.”(Health Expert, P4, Interview4).*

#### Electronic health records and service registration systems

One of the provisions of the PHCEP was electronic health records and service registration systems, which have facilitated the process of diagnosis, care and follow-up of patients.

*“One of the good things was that we record the information in the system”. (CHW, P14, Interview14). “We created electronic files for all the people living in slum areas “. (CHW, P5, Interview5).*

### Threats

The participants pointed out several threats to PHCEP, as shown in Table 5 and explained below.

#### Increasing slum habitation and its socio-economic effects on health

Slum habitations and migration to cities following the increase of urbanization as one of the social factors affecting health is of concern to health policymakers because it endangers the health of the urban population. Health is a multidimensional component and requires multidisciplinary management, despite the implementation of the PHCEP, context factors such as cultural, economic, and social especially in these areas, threatened population health. The study participants agreed on this issue.

*“Increasing the population of slum areas, especially foreign immigrants, is one of the serious threats; their data registration and insurance are challenges too”(University professor, P1, Interview1). “We have a high population turnover in these areas and we are supposed to provide active care, while we have difficulties in identifying the population, and this has made providing services difficult for us. We have a large number of people not registered in the system.”(CHW, P5, Interview5).*

#### Low capacity of the private sector

For reasons such as limited and unequal distribution of resources and low productivity in public health sector, collaborating with private sector can be an opportunity, but this sector has limited experiences in providing primary health services of Iran.

*“I invited the representative of a contracting company, and told him that you should bring maternal care index to this number. He didn’t know what I am saying. He had no experience in this field at all”(Health manager, P12, Interview12). “It is true that the program had many benefits, but the management was not satisfactory; the private partner company did not have power, and the headquarter was involved in all matters, even though it should do the supervision.”(Health manager, P13, Interview13).*

#### Low coordination between service providers and insurance companies

One of the problems of Iran’s health system is the weak coordination of insurance organizations that purchase the services of MOHME as the health service provider. Although the implementation of the universal insurance plan in the vulnerable population was a valuable measure, appointing a physician for these people by an Iranian insurance company outside the area of facility centers was a major challenge for service delivery.

*“There is a wrong process in the insurance company. It chooses a doctor for the insured based on the last time the person visited that doctor and caused problems for our services”(CHW, P5, Interview5). “One of the problems of health system is the lack of coordination between insurance companies and healthcare providers” (Health Expert, P10, Interview10).*

#### Instability of financial resources

Sustainable financing is necessary for the successful implementation of health system programs. The participants considered the lack of stable financial resources as a threat to continues delivery of services.

*“You advise a plan and you don’t have stable financial resources.”(University Professor, P9, Interview9). “We lack necessary resources to continue the system; the program cannot be developed without adequate sustainable resources”. (Health expert, P15, Interview15).*

#### The culture of preferring medical specialists to PHC providers and low awareness and use of PHC services

One of the main problems in providing first-level health services is the desire of the urban community to visit specialists at the beginning of the disease. Introducing preventive service packages to urban society and promoting related culture is essential.

*“One of our challenges that has not been solved is that people tend to visit a specialist in private sector. This issue should be solved and resources can be saved.”(CHW, P5, Interview5) People want second and third level health services instead of PHC. You can’t be successful without primary health centers, now the services are very specialized.”(University professor, P1, Interview1).*

## Discussion

In the present study, the Primary Health Care Expansion Program (PHCEP) from the perspective of the stakeholders was assessed. In this qualitative study, the content analysis method was applied and the framework of the SWOT was used for coding the data. Results of the study showed what strengths and weaknesses the program has and what opportunities and threats it faces**.**

### Strengths

One of the necessary prerequisites for evidence-based planning in health sector and the successful implementation of the programs is information system that includes demographic, environmental and disease burden information. According to the needs and problems, necessary interventions are planned. One of the strong points of this program was context analysis. In a study, Raisi et al. stated that one of the achievements of HSRP in Iran was identifying the characteristics of population and slum areas covered by health centers [24]. Tabrizi et al. stated that one of the strengths of the Health Complexes Model in suburban areas of Iran was identifying the population covered by health service centers [25]. which is in line with the results of the present study.

One of the strong points of this program was the development of physical resources, Before the implementation of HSRP in Iran, one of the main problems at the PHC level was the shortage of physical resources and standard equipment particularly in health posts in cities. With the implementation of the first phase of HSRP in slums, more resources were allocated. According to Abedi et al. one of the key strengths of HSRP was its contribution to the development of healthcare networks [26]. A study in Iran mentioned the development of infrastructure as one of the achievements of HSRP [27]. which confirms the results of our study. Supply of Human Resources, development of centers, processes, service packages and new programs in Iran, needs human resources. One of the important parts of PHCEP was the supply of human resources through private sector and outsourcing of services. A study mentioned PPP as one of the opportunities of the program [24]. Bakhtiari et al. considered health teams working in slums as another important part of health promotion programs [2]. which is consistent with the results of our study.

Strengthening and expansion of PHC services in suburban areas through health team members including doctors, midwives, nurses, public health experts, psychologists, and nutritionists, and delivering packages of services at the first level of health network was one of the strong points of PHCEP program. New packages include nutrition services, mental health, and package of essential non-communicable diseases interventions (Irapen) were implemented. Abedi et al. stated that the implementation of the UFPP with the private sector participation model resulted in expanding the coverage of service packages [16]. Moreover, Jabari et al. explained that service packages and expanding service coverage were among the achievements of the Family Doctor and Health Team Programs, which is in agreement with the findings of our study [28]. Building health teams strengthened and expanded the delivery of New PHC.

One of the strong points of this program was increasing access and equity of health services, because one of the most important goals of PHC services is to create equity in health [29]. Implementation of the program in suburban areas by increasing availability and access to health services, and fair distribution of resources according to people’s needs are examples of equity in providing health services. The results of a study showed that the implementation of FPP and health team programs in cities has increased accessibility [30]. which confirms the results of our study.

### Weaknesses

One of the weaknesses points of this program was type of the outsourcing model in PHCEP, in recent years, outsourcing of health services has increased in developing countries. They aim to improve access, efficiency and quality of services. There are several challenges with regard to outsourcing such as contract models and performance evaluation, which require proper planning [31]. The lack of a suitable model or framework for outsourcing or ambiguity in the contracts with the private sector has been mentioned as a problem in health system [32, 33]. Despite the efforts of MOHME, the lack of integrity in the types and models of outsourcing is a barrier for the process [34]. One of the problems of service outsourcing process is the opportunistic behavior of the private sector [32]. which is in line with the results of our study. Since, one of the main difficulties of PHCEP is outsourcing model of first-level services, it is necessary to carefully choose the services that can be delegated, and then the best outsourcing model.

One of the deficiencies of PHCEP was performance monitoring and evaluation mechanisms. Quality-oriented evaluation was not on the managers’ agenda. According to Eskandari et al., paying too much attention to quantities, reports and ineffective monitoring were the shortcomings of HSRP in slum areas of Qom, Iran [35]. Abedi et al. stated that one of the main shortcomings of UFPP was the lack of a proper monitoring and evaluation system [36]. which confirms the results of our study. In the previous PHC system evaluations, more attention was paid to output indicators than resources and processes. Whether the services are cost-effective or not still is the question. In general, monitoring has taken the place of evaluation. Since, the outsourcing of services is a new experience at the PHC level, the costs and tariff of each service, measurement units, the deductions for delays in providing services, responsible authorities, monitoring and evaluation process, timeline, standard tools and teams should be defined properly.

According to the study participants, human resources management in health facilities of suburban areas was a main challenge. It brought difficulties to outsourcing services such as shortages of human resources, job insecurity, and lack of motivation [32]. The results of a research on the urban UFPP with the public-private sectors partnership model in Iran identified heavy workloads, dissatisfaction, job insecurity and lack of career promotion, welfare facilities, shortages of equipment and service delivery space as the challenges of human resources management [36]. Eskandari et al. showed the decrease in the quality of services due to the increase in workload, incorrect selection of employees, and insufficient training [35]. which are consistent with the results of our study. Because of the vital role of human resources in achieving organizational goals, human resource management is a priority. In PHCEP, using the model of public-private partnership, improving accountability, equality in payments, and incentives, especially for healthcare workers should be considered.

One of the main weaknesses of PHCEP was the payment mechanism. The participants cited the delay in payments and imbalance of payment and workload as problems of the payment mechanism. The results of a study stated that one of the challenges of outsourcing services in health centers was the delay in paying salaries [32]. A study expressed that the imbalance between workload with salary was one of the claims of health team members [36]. lack of suitable payment methods identified as a main challenge facing the UFPP in Iran [37]. Payment mechanism can be a motivational factor for service providers, so it is suggested that in slum areas payment mechanism changes from monthly salaries to a performance-based model. In this model, the level of achieving goals, such as blood pressure control, is the basis for payment not the number of times that blood pressure has been monitored. However, in current situation, this system is not working because the units of services, tariffs and processes have not been determined. One of the main problems of the PHCEP in suburban areas is the referral system and coordination of cares. A study aimed at evaluating the UFPP named the referral system as a main challenge [16, 38]. which is in agreement with the results of our study. Another study on Health Complexes Model in Tabriz, one of the major cities of Iran, expressed that referral clinics and the provision of specialized services were the strengths of the model, which is not consistent with the results of the present study [2]. Patients’ referral is more challenging at PHC level, which is dependent on specialists for consultations, so it is suggested that health policymakers and managers, especially in urban areas pay attention to the establishment of a referral system.

The focus on the design rather than the implementation of the program, not delegating authority to the private sector, and low awareness of the population about PHC have been expressed by the participants as signs of weak project management. A study in the city of Qom, Iran with the aim of explaining the problems of implementing HSRP in slums, stated that health system managers are not open to criticisms; they implement programs without enough preparations; their supervision is not effective, and the quality of services is not optimum [35]. According to another study, constant changes in policies, and internal and external environments, and not delegating authority to the private sector are among the problems of outsourcing health service in Iran, which confirms the results of our study [39]. It is suggested to employ experienced managers and consultants and use evidence-based management to overcome these difficulties.

The participants listed the low efficiency and effectiveness as weak points of PHCEP. The results of a study showed low efficiency of outsourcing services in health facilities [39]. Eskandari et al. stated that the wastage of financial and human resources were problems of HSRP [35]. A study mentioned the lack of proper planning and management for the effective use of resources as problems of HSRP, which is in line with the results of our study [40]. Parts of the problem is related to the outsourcing model, payment mechanism, human resources management and monitoring and evaluation mechanisms. To enhance efficiency, considering them is important.

### Opportunities

One of the opportunities of the program was the support of policy makers and the approved laws for health promotions especially, in the suburban areas. The results of a study showed that service outsourcing in Iran was accompanied by political and legal support particularly 3rd Economic, Social and Cultural Development Plan 1999 [31]. Another study considered the legal support of HSRP as a positive point in providing services, which confirms the results of our study [26]. Since the implementation of PHCEP is supported by policies and laws, program managers need to take this opportunity to strengthen the system and solve its problems.

Building inter-sectoral coordination along with the implementation of the HSRP accounted as an opportunity, that can be facilitated achieving goals of this program. Shortages of resources in public sector, makes using the capacity of health partners necessary. Health partners are not just to provide resources but to collaborate in implementing the program. Many health promotion programs such as the control of non-communicable diseases are facilitated and implemented in cooperation with other organizations. One of the achievements of HSRP was the provision of resources by inter-sectoral coordination and the support of health donors [24]. which is in agreement with the results of our study.

The participation of the private sector in providing health services in suburban areas was considered a very important opportunity by the participants. A study emphasized the importance of private sector in the provision of PHC and stated this sector can improve the performance of health care network [41]. To improve access, efficiency and quality in suburbs of cities, partnership with private sector was suggested, which is consistent with the results of our study [31]. If a proper PPP model is used in Iran, an atmosphere of competition in the supply of health services will be created and the quality of health services will be improved.

The participants called universal health insurance as one of the program’s opportunities for people to use services, including screening, treatment and referral especially for chronic non-communicable diseases. A study stated that one of the achievements of HSRP was the insurance coverage of the population with the aim of improving justice in the provision of health services to the vulnerable population [27].

Employing trained human resources was an opportunity for the program. A study on the urban Family physician program in Iran, implemented in both the public and private sectors, stated that motivated, trained and available manpower is an opportunity, which is consistent with the results of our study [16]. It is necessary for policy makers and managers to improve the mechanisms of recruitment and provide job training to have employees with sufficient motivation, knowledge and skills in PHC centers. Electronic health record system was considered as an opportunity in PHCEP, according to participants’ viewpoints, it has facilitated providing services, recording information, monitoring, and new health services A study showed that electronic record system has brought benefits such as saving time and money, increased the speed of access to services, and satisfaction of employees and people [24]. It is necessary to change the paradigm from quantity to quality in designing electronic health record systems. They should not be just service registration portals (quantity-oriented).

### Threats

From the perspective of the study participants, one of the serious threats to the PHC system is social factors affecting health. Results of a study in Iran stated that urbanization, increasing slum habitations, aging of population, and demographic changes are among the social factors affecting health system [7]. Another study cited the increase in urbanization and changes in lifestyle as reasons for increasing the risk factors of non-communicable diseases [42]. which confirms the results of our study. Considering the increase in the urban population in Iran and the consequent social and economic factors affecting health, service packages should be modified to meet the needs of population, especially in suburban areas.

Although the study participants perceived PPP as a positive prospect, they also noted the sector’s weaknesses in terms of capacity and experience, especially in primary health care. A study aimed at investigating the outsourcing process in Middle East countries including Iran considered inability and low experience of the private sector in providing health services as barriers to PPP [31]. Another study emphasized the low capacity and insufficient experience of this sector in outsourcing health services [39]. Shahrbafchizadeh added the variety of contracts in the PHC sector, and the unfamiliarity of private sector with the services [32]. which is in line with the results of our study. To overcome the unfamiliarity of private companies with the health sector and legal obstacles, training courses should need to be planned for the managers of private companies.

The support of authorities for universal health insurance was seen as a positive opportunity for primary healthcare by the study participants. However, they also highlighted the potential threats of poor coordination and competing programs. The lack of coordination between MOHME programs such as FPP and insurance companies was a main challenge facing the implementation of programs [43]. Therefore, the insurance organizations should coordinate with the MOHME to facilitate providing the services because their coordination will enhance efficiency and effectiveness of programs. From the perspective of the participants, the lack of sustainable financing endangers the timely provision of necessary services. The main challenge of the Health Complexes Model in suburban areas of Tabriz, was instability of financing [44]. which confirm the results of our study. With evidence-based decision making, managers will be able to allocate the resources correctly.

According to the participant’s viewpoints, the culture of preferring medical specialists to PHC providers, and low awareness and use of PHC services are threats to Iran’s health system. A study listed the lack of attention to culture building and health literacy promotion among the barriers to providing health promotion services and implementing FPP [27]. In Health Complexes Model, it was decided to employ a medical specialist for the health promotion team [25]. It seems integrating services and collaborating with specialists in screening and referral of patients will be useful.

## Limitations and strengths

This study encountered a major limitation due to the frequent changes in management within the PHCEP, which made it difficult to conduct interviews with them. Furthermore, the workload of service providers posed challenges in coordinating suitable interview schedules. Despite the limitations. To our knowledge, this qualitative study is the first of its kind to gather feedback from participants at different levels in primary healthcare and employ the SWOT method to evaluate the program.’

## Recommendations for future studies

Future studies should assess the effectiveness of the PPP service delivery model at the PHC level, considering the context and using quantitative methods to gather more evidence. Additionally, we recommend that researchers evaluate the effectiveness of payment mechanisms and their effects on outcomes and the performance of health providers to gain insights and improve the program.

## Conclusion

The results of this study showed the strengths, weaknesses, opportunities and threats of primary health care expansion program implemented with the public-private partnership model in slum areas of Iran. Health policy makers and managers need to pay attention to the internal and external factors affecting health system and interventions. The plans should be in accordance to the context of each province and these strengths, opportunities, weaknesses and threats. Improving payment mechanisms, human resources management, and sustainable financing could help to escalating the program.

### Supplementary Information

**Supplementary Material 1.**

## Data Availability

The datasets generated and analyzed during the current study may be available from the corresponding author upon reasonable request.

## Abbreviations

UHC: Universal health coverage
PHC: Primary health care
MOHME: Ministry of Health and Medical Education
UFPP: Urban family physician program
HSRP: Health sector reform plan
PPP: Public-private partnership
SWOT: The Strengths, Weaknesses, Opportunities and Threats
PHCEP: The Primary Health Care Expansion Program
FPP: Family physician program
