# Supplementary material for: Evaluation of the primary health care expansion program with public-private partnership in slum areas from the perspective of stakeholders: a qualitative study
Source: BMC Prim Care. 2024 Feb 22;25:67. doi: 10.1186/s12875-024-02303-w (PMC10882754; doi:10.1186/s12875-024-02303-w)
Supplement: Supplementary file 1 — Supplementary Material 1. [file 12875_2024_2303_MOESM1_ESM.docx]

_interview guide_

Appendix 1

Evaluation of the Primary Health Care Expansion Program with Public-Private Partnership in slum areas from the perspective of stakeholders: a qualitative study

First part: Interview protocol

1. The time of the interview is determined according to the working conditions and the opinion of the participants.

2.The location of the interview is determined according to the working conditions and the opinion of the participant.

3. The participants are assured that the audio file and interview texts will not be used for purposes other than the research under any circumstances.

4.The participants are assured that the audio file and interview texts will always remain confidential.

5. In order to prevent any bias, the research team provided the transcribes to the participants to confirm after completing the interview.

6.The interviewers make the necessary arrangements to comply with the administrative issues before the interview.

7. The interviewer asks permission from the interviewee to record the audio file.

8. After explaining the objectives of the study and the ethical considerations of the research, the interviewer asks the interviewee to declare her desire to participate in the study in order to record it in the audio file.

Interview questions:

1. Please explain your point of view regarding the program of expanding and strengthening primary health care in urban slum areas with public-private partnership (hereafter: the program)?

2. In your opinion, what are the strengths of the program?

3. In your opinion, what are the weaknesses of the program?

4. In your opinion, to be successful, what opportunities does the program have?

5. In your opinion, what threats does the program face?

Probing questions such as “Is it possible to explain it more”? “What do you mean by this item?”, “Could you please give me an example about that”? and … should be asked in appropriate time and place by interviewer if needed.

Best regard

Research team
